# Development and initial validation of the Bedside Paediatric Early Warning System score

**DOI:** 10.1186/cc7998

**Published:** 2009-08-12

**Authors:** Christopher S Parshuram, James Hutchison, Kristen Middaugh

**Affiliations:** 1 Department of Critical Care Medicine, Hospital for Sick Children, 555 University Avenue, Toronto, Ontario M5G 1X8, Canada; 2 Department of Pediatrics, Hospital for Sick Children, 555 University Avenue, Toronto, Ontario M5G 1X8, Canada; 3 Child Health and Evaluation Sciences Program, The Research Institute, Hospital for Sick Children, 555 University Avenue, Toronto, Ontario M5G 1X8, Canada; 4 Centre for Safety Research, Hospital for Sick Children, 555 University Avenue, Toronto, Ontario M5G 1X8, Canada; 5 Department of Pediatrics, University of Toronto, 27 King's College Circle, Toronto, Ontario, M5S 1A1, Canada; 6 Interdepartmental Division of Critical Care Medicine, University of Toronto, 27 King's College Circle, Toronto, Ontario, M5S 1A1, Canada; 7 Department of Health Policy Management and Evaluation, University of Toronto, 27 King's College Circle, Toronto, Ontario, M5S 1A1, Canada

## Abstract

**Introduction:**

Adverse outcomes following clinical deterioration in children admitted to hospital wards is frequently preventable. Identification of children for referral to critical care experts remains problematic. Our objective was to develop and validate a simple bedside score to quantify severity of illness in hospitalized children.

**Methods:**

A case-control design was used to evaluate 11 candidate items and identify a pragmatic score for routine bedside use. Case-patients were urgently admitted to the intensive care unit (ICU). Control-patients had no 'code blue', ICU admission or care restrictions. Validation was performed using two prospectively collected datasets.

**Results:**

Data from 60 case and 120 control-patients was obtained. Four out of eleven candidate-items were removed. The seven-item Bedside Paediatric Early Warning System (PEWS) score ranges from 0–26. The mean maximum scores were 10.1 in case-patients and 3.4 in control-patients. The area under the receiver operating characteristics curve was 0.91, compared with 0.84 for the retrospective nurse-rating of patient risk for near or actual cardiopulmonary arrest. At a score of 8 the sensitivity and specificity were 82% and 93%, respectively. The score increased over 24 hours preceding urgent paediatric intensive care unit (PICU) admission (*P *< 0.0001). In 436 urgent consultations, the Bedside PEWS score was higher in patients admitted to the ICU than patients who were not admitted (*P *< 0.0001).

**Conclusions:**

We developed and performed the initial validation of the Bedside PEWS score. This 7-item score can quantify severity of illness in hospitalized children and identify critically ill children with at least one hours notice. Prospective validation in other populations is required before clinical application.

## Introduction

Clinical deterioration resulting in near or actual cardiopulmonary arrest in hospitalised children is common [1], associated with adverse outcome [2,3] and may be preventable [4-7]. Timely identification and referral of children may be facilitated by the application of calling criteria or severity of illness scores. The major limitation of available severity of illness scores for hospitalised patients is complexity [4,8,9]. Complex scores are not feasible to implement at the bedside, limiting their ability to function as real-time instruments to improve patient safety [8,10].

Our group previously developed a 16-item severity of illness score for use in hospitalised children [4]. It had favourable performance characteristics; however, its complexity was felt to limit clinical application [10]. The objective of this study was to create a simple score for routine bedside use. The purpose of this score was to quantify severity of illness across in hospitalised children. We wanted the score properties of the new score to include a range of scores between 'sick' and 'well' patients to permit the future development of score-matched care recommendations. We called this new score the Bedside Paediatric Early Warning System (PEWS) score.

## Materials and methods

The Bedside PEWS score was developed and initial validation was performed. The goal of score development was to create a simple severity of illness score that could discriminate between sick and less sick children for use as part of routine care. Validation of the Bedside PEWS score involved evaluations comparing the score versus expert opinion, progression of the score over time, and the scores and outcomes of children referred to, or followed by a Paediatric Medical Emergency Team, called the Critical Care Response Team (CCRT).

### Clinical data

Study data were obtained from three sources: patients in a case-control study, a survey of nurses caring for the patients in the case-control study, and prospectively collected data from patients seen by the CCRT.

Eligible patients for the case-control study were admitted to a hospital ward at the Hospital for Sick Children, had no limitations to their care and were less than 18 years of age. 'Case' patients were admitted urgently to the paediatric intensive care unit (PICU) from a hospital inpatient ward following urgent consultation with the PICU, but not following a call for immediate medical assistance (a 'code-blue' call). 'Control' patients were admitted to an inpatient ward (not the PICU, neonatal ICU, an outpatient area or the emergency department) during the period of study, and in the 48 hours following inclusion did not have a 'code-blue' call and were not urgently admitted to the PICU. Case patients were identified by prospective daily screening of PICU admissions; control patients were frequency matched with each case patient on the basis of age group, and the type of ward. Two control patients were recruited for each case patient.

Clinical data were abstracted directly from the medical record and was supplemented by interview with consenting frontline nursing staff. Data was collected for 12 hours in control patients, and for 24 hours ending at the time of urgent PICU admission in case patients. The study nurses recorded the clinical data that was documented and that which was not documented but was known by the frontline nurses. They did not calculate candidate scores or sub-scores. Nurses completed a survey describing the number of patients they were looking after, their years of post-graduate experience, and asking 'how surprised would you have been if your patient had a patient care emergency while you were on your break?' on a five-point scale from 'extremely surprised' to 'not at all surprised'. We used this retrospective question to measure the respondent's perception about the child's risk of near or actual cardiopulmonary arrest at the time the child was in the responding nurse's care.

From the prospectively documented CCRT data, we abstracted the items of the Bedside PEWS score, the nature of the consultation and the disposition of the patient following each consultation episode. New consultation episodes included the initial consultation visit and visits over the subsequent 24 hours. Post-ICU discharge review is a mandated activity of the CCRT. Post-ICU discharge episodes included all visits in the two days following ICU discharge. Data from CCRT patients was collected from 1 May to 31 December, 2007.

### Score development

The development of the Bedside PEWS score involved the identification and selection of items that were part of routine clinical assessment and exclusion of demographic and other fixed items from our previously published score [4]. Selected items were modified using the opinions of experienced respiratory therapists, nurses and physicians to define new cut-off points and additional severity categories for candidate items. These candidate items were then evaluated singly and then in combination for inclusion in the Bedside PEWS score using a frequency-matched case-control design.

#### Item reduction

Item reduction occurred in a two-stage process. First, item selection was based on the ability of each item to discriminate between sick and well children. The area under the receiver operating characteristics curve (AUCROC) was used to categorise each item [11]. Items with an AUCROC of 0.65 or less or with a non-significant (*P *≥ 0.05) difference between the mean maximum score were excluded. The remaining items were then stratified into two groups; core items with AUCROC above 0.75 were included in the score. Items with AUCROC of 0.75 or less were ranked on the basis of the difference between maximum sub-scores and the frequency of measurement. The frequency of measurement for each candidate item was expressed as a proportion of the total number of times that one or more measurements were documented or known by the frontline nurse. The intermediate items were added to the core items to create a list of candidate scores.

Second, the performance of candidate scores was evaluated. For each alternate score, the mean and maximum scores were determined for each patient. The maximum score for each patient was used to reflect the worst clinical condition. The AUCROC for each candidate score was determined using the maximum Bedside PEWS score over 12 hours in control patients, and from the 12 hours ending 1 hour before ICU admission in case patients. Scores with greater AUROC were chosen preferentially over those with lower areas. Candidate scores with greater differences between scores for case and control patients were chosen over scores with smaller differences in combination with clinical judgement.

### Score validation

Following development of the Bedside PEWS score, we evaluated its convergent validity, responsiveness and construct validity.

We hypothesised that Bedside PEWS scores were (1) correlated with nurse-rated risk of near or actual cardiopulmonary arrest, (2) higher in the children who were urgently referred for ICU consultation versus following ICU discharge, (3) higher in children who were admitted urgently to the ICU than in other patients for whom the ICU was urgently consulted, and (4) that Bedside PEWS scores increased over the 24 hours preceding ICU admission.

We compared the Bedside PEWS scores in patients with new consultation and following ICU discharge by the outcome of consultation (ICU admission or not). Finally, for all visit episodes not resulting in ICU admission we compared the Bedside PEWS scores with the time to the planned follow-up visit. We excluded visits where the follow-up plans were not indicated. The frontline staff of the CCRT were not familiar with the Bedside PEWS score, the score was not calculated, and was not used to assist in management, disposition or follow-up decisions.

### Analyses and data management

Data was entered into an Oracle Database (Redwood Shores, CA, USA). The accuracy of data accuracy was verified by independent manual comparison of all entered data with the case report forms and electronic evaluation for internal consistency. When inconsistencies could not be resolved from the case report form, the original medical record was reviewed.

Clinical data was grouped into one-hour blocks for 24 hours ending at PICU admission in cases or the end of 12 hours data collection in controls. The greatest sub-score for each item in each hour was identified and was used to calculate the Bedside PEWS score for each hour. Logistic regression was used to evaluate the performance of individual items and candidate scores. The AUCROC was determined from the c statistic calculated by the logistic procedure.

The maximum scores for control versus case patients were compared by t-test and regression analysis. The maximum PEWS score was calculated for the time intervals: in four-hour blocks relative to ICU admission, over the time described by each nursing survey; for the 12-hour period of the case-control study; and at the point of initial contact of the ICU follow up or urgent referral.

The case-control status was then used as the dependent variable in logistic regression analyses. The primary analysis compared the maximum Bedside PEWS in cases and controls. Next, we compared case-control status with nurse rating of risk of near or actual cardiopulmonary arrest, and then used a multivariable model to evaluate relations with the maximum Bedside PEWS score (for the 12 hours of the case-control study), the nurse rating of patient risk, nurse experience and the nurse-patient ratio. Backwards elimination removed variables until only those present at the *P *< 0.05 level remained in the model.

A correlation analysis was used to evaluate the relation between the maximum Bedside PEWS score and nurse rating of risk of near cardiopulmonary arrest for the time period that the rating nurse cared for the patient. The maximum Bedside PEWS score was then used as the dependent variable in regression analyses. First, a random co-efficients mixed model regression compared the mid-point of the time interval with the maximum Bedside PEWS score from that interval. Next, we included the square of the mid-point of the hour in this regression. Third, a multi-variable linear regression compared the maximum Bedside PEWS score (for the 12 hours of the case-control study) with case-control status, the nurse-patient ratio and nurse experience. Nurse experience from the survey (<0.5, 1 to 5 years, >5 years) was conservatively represented as 0.5, 2.5 and 5 years, respectively. A backward elimination process was used. The r^2 ^was used as a measure of the variability in the maximum Bedside PEWS score that was explained by the variables evaluated.

For patients seen by the CCRT we obtained data from our hospital's patients in the provincial database. For each patient visit we calculated the Bedside PEWS score. Where the available data permitted the calculation of more than one score per patient visit we calculated both and used the greatest score for analysis. For patients seen in a new consultation we compared the Bedside PEWS score for the initial consultation visit with the disposition of the patient over the next 24 hours. Patients who were classified as either: admitted to the ICU (1) as part of the initial consultation, (2) after the initial consultation and within the next 24 hours, or (3) as not admitted within the first 24 hours of consultation. Comparisons were made using analysis of variance.

The Bedside PEWS scores of patients who were seen by the CCRT were compared by the disposition of the patient using a Student's t-test. The time to planned follow up was tabulated. Linear regression was used to compare the Bedside PEWS score with the mid-point of the time-interval for the planned follow up category.

Data management and analyses were performed using SAS v 9.2 the power to know™ (Cary, NC, USA). A *P *value of less than 0.05 was regarded as significant. The protocol was reviewed and approved by the Research Ethics Board at the Hospital for Sick Children (REB approval 1000004218). Consent was required from nurse participants, but not from patients, parents or their surrogates.

## Results

### Clinical data

Candidate items and scores were evaluated in clinical data from 60 urgent ICU admissions and 120 well control patients (Table 1). The mean age of children studied was 72 months, and was comprised of 32 children aged younger than 3 months; 35 children aged 3 to 12 months; 22 children aged 1 to 4 years; 54 children aged 5 to 12 years and 37 children aged older than 12 years. Measurements of clinical data were made at 2961 individual times. The most frequently measured items were heart rate, respiratory rate respiratory effort, oxygen therapy and level of consciousness (Table 2).

**Table 1:** Candidate items evaluated for Bedside PEWS score

Item		Item sub-score			
	Age group	0	1	2	4

Heart rate	0–3 months	>110 and <150	≥ 150 or ≤ 110	≥ 180 or ≤ 90	≥ 190 or ≤ 80
	3–12 months	>100 and <150	≥ 150 or ≤ 100	≥ 170 or ≤ 80	≥ 180 or ≤ 70
	1–4 years	>90 and <120	≥ 120 or ≤ 90	≥ 150 or ≤ 70	≥ 170 or ≤ 60
	4–12 years	>70 and <110	≥ 110 or ≤ 70	≥ 130 or ≤ 60	>150 or ≤ 50
	>12 years	>60 and <100	≥ 100 or ≤ 60	≥ 120 or <50	≥ 140 or ≤ 40
Systolic blood pressure	0–3 months	>60 and <80	≥ 80 or ≤ 60	≥ 100 or ≤ 50	≥ 130 or ≤ 45
	3–12 months	>80 and <100	≥ 100 or ≤ 80	≥ 120 or ≤ 70	≥ 150 or ≤ 60
	1–4 years	>90 and <110	≥ 110 or ≤ 90	≥ 125 or ≤ 75	≥ 160 or ≤ 65
	4–12 years	>90 and <120	≥ 120 or ≤ 90	≥ 140 or ≤ 80	≥ 170 or ≤ 70
	>12 years	>100 and <130	≥ 130 or ≤ 100	≥ 150 or ≤ 85	≥ 190 or ≤ 75
Capillary refill		<3 sec			≥ 3 sec
Pulses		Normal	Weak	Doppler or bounding	Absent
Bolus fluid		No	Yes		
Respiratory	0–3 months	>29 and <61	≥ 61 or ≤ 29	≥ 81 or ≤ 19	≥ 91 or ≤ 15
rate	3–12 months	>24 or <51	≥ 51 or ≤ 24	≥ 71 or ≤ 19	≥ 81 or ≤ 15
	1–4 years	>19 or <41	≥ 41 or ≤ 19	≥ 61 or ≤ 15	≥ 71 or ≤ 12
	4–12 years	>19 or <31	≥ 31 or ≤ 19	≥ 41 or ≤ 14	≥ 51 or ≤ 10
	>12 years	>11 or <17	≥ 17 or ≤ 11	≥ 23 or ≤ 10	≥ 30 or ≤ 9
Respiratory effort		Normal	Mild increase	Moderate increase	Severe increase/any apnoea
Saturation		>94	91–94	≤ 90	
Oxygen therapy		Room air		Any – <4 L/min or <50%	≥ 4 L/min or ≥ 50%
Level of consciousness		Normal Consolable Rouseable Bromage 0,1,S			Bromage score 2–3 Irritable
Temperature °C		≥ 36 and ≤ 38.5	<36 or >38.5	<35 or >40	

Candidate items for the Bedside Paediatric Early Warning System (PEWS) score are presented. Expert opinion was used to identify cut-off points for scoring each item. Item values that fall in the stated ranges receive the number of points indicated at the top of each column. For example a 13-year-old with a respiratory rate of >11 and <17 breaths per minute will receive 0 respiratory sub-score points, whereas if the respiratory rate was either <9 or >30 breaths per minute then 4 sub-score points would be assigned. Given the limitations of assessment and documentation we were unable to use the Glasgow coma scale as the primary measure of level of consciousness. Consequently, level of consciousness was assessed with the Bromage Sedation Scale and an infant behaviour description used locally. The Bromage Sedation Scale is 0 – awake, 1 – occasionally drowsy, easily rouseable, 2 – frequently drowsy, easily rouseable, 3 – somnolent, difficult to arouse and S – normal sleep. The infant behaviour scale was adapted from local documentation practice to describe a child who was irritable, rouseable, consolable, or 'normal'. These two categories were combined to describe level of consciousness. A Bromage score of 2 or more or an infant behaviour rating of 'irritable' was assigned 4 sub-score points.

**Table 2:** Frequency of measurement and item sub-scores of candidate items for Bedside Paediatric Early Warning System score

All patients		Controls		Urgent ICU admission				
Item	Proportion of times with measurements n/N	Mean maximum subscore	Proportion of observation times with measurements	Mean maximum subscore	Proportion of observation times with measurements	Difference of means	*P*	AU ROC

HR	69.6%	0.87	52.9%	2.45	89.9%	1.58	<0.001	0.814
SBP	33.1%	0.78	23.0%	1.52	45.5%	0.74	<0.001	0.670
CRT	27.4%	0.50	22.3%	1.93	33.6%	1.43	<0.001	0.679
Pulse	18.7%	0.04		0.46		0.42	<0.001	0.627

RR	48.9%	0.64	32.0%	2.00	69.5%	1.36	<0.001	0.795
Respiratory effort	70.5%	0.20	81.7%	1.77	56.9%	1.56	<0.001	0.786
Saturation	65.1%	0.45	45.3%	1.18	89.2%	0.73	<0.001	0.677
Oxygen therapy	92.9%	0.40	92.3%	2.47	93.6%	2.07	<0.001	0.835

Bolus	13.1%	0.03	10.4%	0.11	12.9%	0.08	0.067	0.542
Temperature	25.2%	0.10	21.5%	0.55	0.323	0.45	<0.001	0.697
						0.000		
Infant behaviour scale	8.6%	0.900	5.2%	1.40	14.8%	0.500	0.075	0.563
Bromage sedation	79.5%	0.000	90.6%	0.67	60.4%	0.670	<0.001	0.583
Level of Consciousness	88.1%	0.900	94.8%	1.93	83.8%	1.033	0.004	0.629

We present a description of the candidate items and their performance as discriminators between 60 case patients who were urgently admitted to the intensive care unit (ICU; without 'code blue' event) and 120 control patients who had neither urgent ICU admission nor code blue event. The first, third and fifth columns show the number of measurements expressed as a percentage of the number of times that any clinical measurement was made. For example heart rate (HR) measurements were recorded on 79.6% of occasions when any clinical measurement was made. For each candidate item the mean of the maximum sub-scores for case patients and control patients is presented. The maximum sub-score value from each patient was used in logistic regression to calculate the area under the receiver operating characteristics curve (AUROC) for each candidate item. The difference between these measurements is presented in the column titled difference of means and the *P *value represents the comparison between case and control patients using logistic regression. CRT = capillary refill time in seconds, RR = respiratory rate; SBP = systolic blood pressure.

### Score development

Eleven candidate items were evaluated; heart rate, systolic blood pressure, capillary refill time (CRT), pulses, bolus fluid administration, respiratory rate, respiratory effort, trans-cutaneous oxygen saturation, oxygen therapy, level of consciousness and temperature. Given the infrequent scoring with the Glasgow Coma Scale we found in our previous work, the Bromage Sedation Scale and a description of infant behaviour was used to assess levels of consciousness [12]. Expert-derived categories were associated with sub-scores of 0, 1, 2 or 4 (Table 1) for each item.

### Item selection

Sub-scores from 10 of 11 items were significantly different (all *P *< 0.0001) with differences between case and control patients ranging from 0.42 to 2.0 points (Table 2). Sub-scores were not significantly different between case and control patients for bolus fluid administration (*P *= 0.07), and this item was excluded from further evaluation.

The AUCROC for the remaining items ranged from 0.54 to 0.83 (Table 2). Heart rate, respiratory rate, respiratory effort and oxygen therapy had AUCROC of more than 0.75 and were included in the score. Level of consciousness and pulses did not adequately discriminate and were excluded from further evaluation (AUCROC ≤ 0.65).

There were four remaining candidate items with intermediate AUCROC of more than 0.65 and 0.75 or less. These items were measured with differing frequencies and had differences between maximum sub-scores for case and control patients; systolic blood pressure (33%, 0.74), saturation (61%, 0.73), CRT (25%, 1.4), and temperature (25%, 0.45), suggesting that CRT had the greatest potential impact on the total score, and temperature the least.

Four candidate scores were then evaluated. The simplest was the four core items. CRT was added to the core items, followed by the addition of saturation and then systolic blood pressure. Temperature was added as the last item.

### Performance of candidate scores

All candidate scores could discriminate between case and control patients. Scores containing more items had greater maximum and mean scores, and greater differences between groups (Table 3). The difference between the mean maximum scores of case and control patients ranged from 5.8 in the core item only score, to 6.9 in the score with all eight items. The inclusion of temperature did not greatly alter the AUCROC, maximum or mean scores of case and control patients, and it was excluded. The addition of systolic blood pressure and transcutaneous oxygen saturation to the four core items with capillary refill increased the mean score by 0.73 in cases and the difference between the mean score of cases and control patients by 0.37 (Table 3). These differences were judged to be clinically important and consequently seven items were included in the Bedside PEWS score. These items were: heart rate, systolic blood pressure, CRT, respiratory rate, respiratory effort, transcutaneous oxygen saturation and oxygen therapy.

**Table 3:** The performance of alternate scores

Score		Mean score			Maximum score			
Composition	*Range*	WELL	PICU*	Difference	WELL	PICU*	Difference	AUROC (95% CI)

Core items	0–16	0.91	4.56	3.66	2.01	7.82	5.81	0.91 (0.86–0.96)
Core + CRT	0–20	1.04	5.05	4.01	2.47	8.95	6.48	0.91 (0.86–0.96)
Core + CRT + Satn + SBP	0–26	1.39	5.78	4.39	3.38	10.08	6.70	0.91 (0.86–0.96)
All 8 items	0–30	1.40	5.86	4.46	3.43	10.31	6.88	0.92 (086–0.97)

The table represents the evaluation of candidate scores. All scores include four core items that discriminated between sick (PICU) and well with an area under the receiver operating characteristics curve of >0.75. These items were heart rate, respiratory rate, respiratory effort and oxygen therapy and are designated as 'core items'. We added the candidate items capillary refill time (CRT), transcutaneous oxygen saturation (Satn) and systolic blood pressure (SBP) to the core items and last evaluated these seven items plus temperature. AUROC = area under the receiver operating characteristics curve; CI = confidence interval.

**Figure 1:**
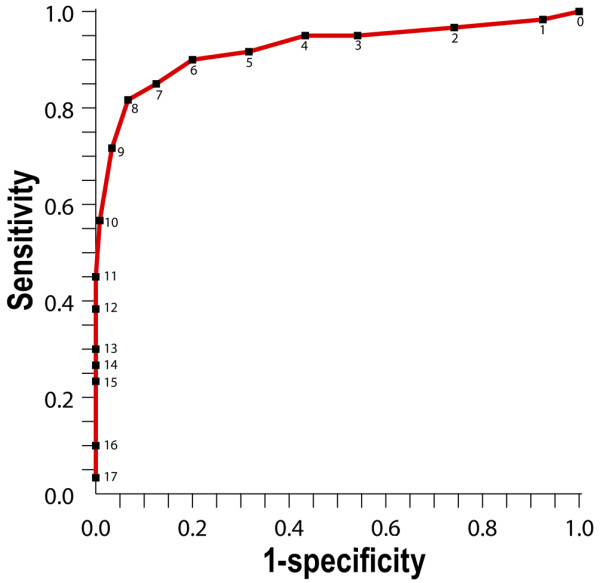
The receiver operating characteristics curve for the maximum Bedside Paediatric Early Warning System score. Results are shown for the 11 hours ending one hour before urgent ICU admission and for 12 hours in control patients who had not clinical deterioration event. The area under the receiver operating characteristics curve was 0.91 with sensitivity 82% and specificity 93% at a threshold score of 8.

The maximum possible Bedside PEWS score is 26 and the minimum 0. The mean maximum score in case patients was 10.1 and the difference between the mean maximum scores of control and case patients was 6.7. The AUCROC was 0.91 with sensitivity 82% and specificity 93% at a threshold score of 8 (Figure 1).

### Validation

#### Comparison with retrospective nurse perceptions

Frontline nurses completed 226 surveys describing severity of illness in 168 (93%) patients, with a median of 1 (interquartile range (IQR) 1 to 2) surveys completed per case patient and 1 (1 to 1) per control patient. All nurses who were contacted consented to participate. The maximum PEWS score within the time that the surveyed nurse cared for the patient was positively correlated with their perception of the risk of clinical deterioration near or actual cardiopulmonary arrest (r = 0.536, *P *< 0.0001). The correlation between the maximum Bedside PEWS score and the nurse rating of risk of near or actual cardiopulmonary arrest was -0.26 for controls (*P *= 0.0037), and was not significantly different from zero in case patients (*P *= 0.9986). The multi-variable regression analysis of the maximum score sequentially removed nurse experience (*P *= 0.82, r^2 ^= 0.49), nurse patient ratio (*P *= 0.72, r^2 ^= 0.49) and nurse rating of patient risk of near or actual cardiopulmonary arrest (*P *= 0.06, r^2 ^= 0.51), leaving the case-control status (*P *< 0.0001, r^2 ^= 0.49) as the only factor significantly associated with the maximum Bedside PEWS score. The interaction term with nurse experience and rating of patient risk of near or actual cardiopulmonary arrest was not significant.

In a logistic regression the case-control status was significantly associated with the retrospective nurse rating of patient risk of near or actual cardiopulmonary arrest (*P *< 0.0001, AUCROC 0.84). Multi-variable logistic regression found three variables were significantly associated with case-control status: the maximum Bedside PEWS score (*P *< 0.0001), the nurse-patient ratio (*P *= 0.028), and the nurse rating of the child's risk of near or actual cardiopulmonary arrest (*P *= 0.0005). The AUCROC was 0.94.

#### Responsiveness to changing clinical condition

**Figure 2:**
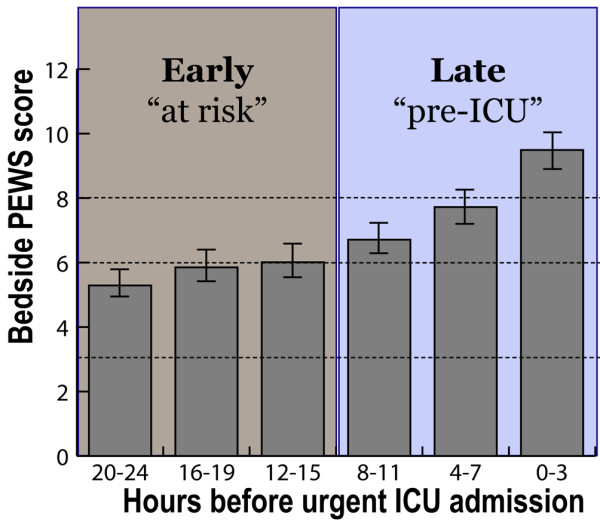
Progression of Bedside PEWS score with increasing proximity to urgent paediatric ICU admission. We present the mean of the maximum Bedside Paediatric Early Warning System (PEWS) score and standard error of the mean for time periods 0–3, 4–7, 8–11, 12–15, 16–19 and 20–24 hours before intensive care unit (ICU) admission.

The maximum Bedside PEWS score increased with increasing proximity to ICU admission from mean maximum scores of 5.3 to 6.0 more than 12 hours before PICU admission to 9.5, 0 to 3 hours before PICU admission (*P *< 0.0001, Figure 2). The square of the mid-point of the hour was also associated with the score (*P *= 0.005).

#### Bedside PEWS scores of patients after ICU discharge and with urgent ICU consultation

There were 436 urgent CCRT consultation episodes for 309 patients (Table 4); 126 (29%) patient-episodes resulted in ICU admission within 24 hours of consultation. Patients who were urgently admitted had higher maximum Bedside PEWS scores (median 7 vs 4, *P *< 0.0001) than patients who were not admitted. The Bedside PEWS scores from the initial visit were greater in patients who were admitted to ICU on the initial visit than those who were admitted later (median 7 vs. 5, *P *= 0.048).

**Table 4:** Inpatients with urgent consultation to the critical care response team

	All visits		Visits with ≥ 5 Bedside PEWS items	
New consults	N (%)	Mean (SD) First visit score	N (%)	Mean (SD) First visit score

Admitted				
On first visit	75 (17%)	7.7 (5.0)	63 (16%)	8.7 (4.7)
Within 24 hours after first visit	51 (12%)	5.9 (3.2)	47 (12%)	6.1 (3.2)
Not admitted	310 (70%)	4.9 (3.4)	272 (71%)	5.2 (3.3)

Data are from 436 urgent consultation episodes to the critical care response team, made for 309 inpatients. Each consultation episode was defined as the 24-hour period beginning at the time of consultation. The outcome of each consultation episode was either remaining on the ward or being admitted to the intensive care unit (ICU). Bedside Paediatric Early Warning System (PEWS)scores were calculated from data collected by the critical care response team (on their arrival or during their consultation). The Bedside PEWS scores of patients who were admitted on the first consultation visit were higher than the scores than patients who were admitted later within the 24 hours of consultation (*P *= 0.048), and than the scores of patients who were not admitted to ICU (*P *< 0.0001). SD = standard deviation.

There were 2975 patient visits performed for the 977 ICU discharge episodes. The median (IQR) Bedside PEWS score was 2 (1 to 4). The 15 patients who were re-admitted to the PICU had higher Bedside PEWS scores 8 (5 to 11) than patients who were not admitted (*P *< 0.0001).

There were 4501 patient-visits made by the CCRT that did not result in urgent ICU admission. The Bedside PEWS scores were greater in patients who had shorter time to next planned review. The proportion of episodes with Bedside PEWS scores of 8 or more, decreased from 24.5% in patients who were to be reviewed within four hours, to 0.5% of patients to be reviewed in 24 to 48 hours (Table 5).

**Table 5:** Planned review times for all patients remaining on ward after critical care response team consultation

Follow up planned	N	Median (IQR)	Mean (SD)	Score of ≥ 8 N (%)
<4 hours	412	5 (3–7)	5.2 (3.3)	101 (24.5%)
4–12 hours	585	4 (2–6)	4.3 (2.8)	81 (13.8%)
12–24 hours	2118	2 (1–4)	2.9 (2.3)	97 (4.6%)
24–48 hours	408	2 (1–3)	2.3 (2.0)	11 (2.7%)
None	963	2 (1–3)	2.3 (2.0)	21 (2.2%)

The table represents the time to the next planned follow up visit for 4501 patient visit episodes by a critical care response team. Hospital patients reviewed included patients for whom an urgent consultation was requested and for patients following discharge from the intensive care unit. Bedside Paediatric Early Warning System (PEWS) scores were calculated from data collected by the critical care response team (on their arrival or during their consultation). The Bedside PEWS scores were higher in patients with shorter times to next review (*P *= 0.034). IQR = interquartile range; SD = standard deviation.

## Discussion

We describe the development and initial validation of the Bedside PEWS score. We reviewed 11 items, removed four, and created a seven-item score to quantify severity of illness in hospitalised children. The seven items in the Bedside PEWS score are heart rate, systolic blood pressure, CRT, respiratory rate, respiratory effort, transcutaneous oxygen saturation and oxygen therapy. These four respiratory and three circulatory variables can be objectively measured in children who are awake and asleep, do not require laboratory or other diagnostic testing, suggesting that the Bedside PEWS score may be feasibly used in clinical practice. The score items have face validity and modest overlap with severity of illness scores for critically ill children in ICUs and emergency departments [13-17].

We found that the Bedside PEWS score can differentiate between hospitalised children with and without critical illness (AUCROC 0.91). This is at least equivalent to more complicated scores [4,8,15]. Using a threshold score of 8, the Bedside PEWS score could identify more than 80% of patients who were urgently admitted to the PICU with at least one hours notice. This compares favourably with our earlier, more complicated, 16-item score [4].

Several additional findings suggest that the Bedside PEWS is a good measure of severity of illness. First, the Bedside PEWS score increased over time leading up to ICU admission. This finding is consistent with observations in other populations, [18] and indicates the Bedside PEWS score is responsive to changes in clinical condition over time in patients – specifically the clinical deterioration associated with evolving critical illness (Figure 2). Scores were greatest in the last 12 hours before urgent ICU admissions. Scores in the 12 to 24 hours before urgent ICU admission were 5.3 to 6.0, values that were higher than we found in 'well' control patients.

Second, the ability of the Bedside PEWS score to prospectively distinguish critically ill from well patients was as good – if not superior to – the retrospective opinion of the bedside nurses who cared for these patients (AUCROC 0.84). The inclusion of both nurse rating and the Bedside PEWS score increased the AUCROC from 0.91 to 0.94. These data suggest that the Bedside PEWS score may provide objective real-time data to compliment frontline provider knowledge, and to better inform level of care and management decision-making [19-21].

Third, the time to the planned review of patients seen by the ICU team is a prospectively articulated marker of the risk of clinical deterioration manifest as near or actual cardiopulmonary arrest. We found patients with higher Bedside PEWS scores had shorter time to planned review (*P *= 0.034). Concordance between the Bedside PEWS score and the prospective management plan of a team with critical care expertise further suggests that the Bedside PEWS score is a good measure of severity of illness.

### Implications for the use of Bedside PEWS

Our data suggest that early identification of patients with evolving critical illness by the Bedside PEWS may permit the targeted application of intermediate response strategies (increased intensity of observation and management), mitigate clinical deterioration and prevent ICU admission, rather than waiting for a 'trigger' to call the ICU team for urgent pre-arrest management [5,22]. Previous experience from the negative cluster randomised trial of medical emergency teams underscores the importance of appropriate mechanisms to identify patients at risk. In this study of 120,000 patients, less than half of patients who had a cardiac arrest, unplanned ICU admission or unexpected death, met calling criteria more than 15 minutes before their event [23]. In contrast more than 80% of patients were identified with at least one hours notice in this study of the Bedside PEWS score.

Evaluation of the Bedside PEWS development dataset shows that a score of 8 offers the best combination of sensitivity and specificity, and provides a statistical basis for recommending a threshold for ICU admission. Evaluation of data from the CCRT, suggests that the application of ICU expertise to patients before possible ICU admission may limit the value of this threshold as for ICU admission, and that this level may be better viewed as a threshold for ICU consultation. Nearly 25% of the consultation episodes resulting in review within four hours were for patients with scores of 8 or more (Table 5). Conversely, 25% of the patients for whom the CCRT was urgently consulted, had scores of two or less (Table 4). We did not assess the appropriateness of consultation; however, it seems reasonable to suggest that many urgent requests for CCRT consultation may have been avoided with the prospective application of the Bedside PEWS score.

### Limitations

There are several limitations to this study. First, the results of his single-centre study may not generalise to other settings or populations. Prospective validation in different settings and with other patient populations is needed. Second, the clinical data contained many missing values. Ideally, complete data would have been prospectively obtained. To reduce the effect of missing data, we asked nurses to recall clinical data they observed but did not document, and we grouped data into one-hour blocks for score calculation. Despite this, prospective scoring of all seven items may have resulted in more complete data and higher scores than we found. The introduction of vital sign-based detection systems may increase documentation [24]. Third, the accuracy of data abstraction was not assessed, against either prospectively collected data, or by repeated assessment. Fourth, we did not evaluate children for whom an immediate call for medical assistance to treat near or actual cardiopulmonary arrest was made. These children may be systematically different than patients who are recognised and admitted urgently to the ICU. Further validation in this and other populations is required before clinical application.

## Conclusions

We describe the development and initial validation of the Bedside PEWS score. This seven-item score increased over the time leading up to urgent ICU admission, provided additional information to compliment retrospective nurse-rated of risk of sudden deterioration, and was higher in children who were subsequently admitted to the PICU than in 'well' control children. Taken together, these data suggest that the Bedside PEWS can quantify severity of illness in hospitalised children. Following successful validation in other populations, clinical application of the Bedside PEWS may facilitate early identification of patients at risk, permitting timely intervention to prevent clinical deterioration, preventing unnecessary ICU admission and acquired morbidity to improve the outcomes of hospitalised children.

## Key messages

• The Bedside PEWS Score is a simple, seven-item severity of illness score for hospitalised children. Scores range from 0 to 26.

• The Bedside PEWS Score can differentiate sick from well patients and identify more than 80% of patients with at least one hours notice before urgent ICU admission.

• As a tool to discriminate between sick and well children, the Bedside PEWS Score was superior to the retrospective opinion of frontline nurses, and was similar to both the score and nurse opinion combined.

• The actions of an ICU-based medical emergency team were concordant with the Bedside PEWS Scores. Higher scores were associated with ICU admission and more frequent secondary review.

## Abbreviations

AUCROC: area under the receiver operating characteristics curve; CCRT: Critical Care Response Team; CRT: capillary refill time; IQR: interquartile range; PEWS: Paediatric Early Warning System; PICU: paediatric intensive care unit.

## Competing interests

CP and JH received funding from The Heart and Stroke Foundation of Canada. KM received salary as the Bedside PEWS research nurse co-ordinator. CP and KM are named inventors of a patent on the Bedside Paediatric Early Warning System that is owned by the Hospital for Sick Children.

## Authors' contributions

CP was responsible for conception and design, analysis and interpretation of data, drafted the manuscript and was involved in critical revisions for important intellectual content. KM was responsible for conception and design, data collection, interpretation of analysis and was involved in critical revisions for important intellectual content of the manuscript. JH was in part responsible for conception and design, and was involved in critical revisions of the manuscript for important intellectual content. Each author has given final approval of the version to be published.
